# Supplementary material for: Zyxin regulates embryonic stem cell fate by modulating mechanical and biochemical signaling interface
Source: Commun Biol. 2023 Jan 18;6:62. doi: 10.1038/s42003-023-04421-0 (PMC9849324; doi:10.1038/s42003-023-04421-0)

Supplementary Information

Zyxin regulates embryonic stem cell fate by modulating mechanical and biochemical signaling interface

Songjing Zhang[1], Lor Huai Chong[2,3], Jessie Yong Xing Woon[1], Theng Xuan Chua[1], Elsie Cheruba[4], Ai Kia Yip[2], Hoi-Yeung Li[1], Keng-Hwee Chiam[2*], Cheng-Gee Koh[1*]

[1]School of Biological Sciences, Nanyang Technological University, Singapore

[2]Bioinformatics Institute A*STAR, Singapore

[3]School of Pharmacy, Monash University Malaysia, Subang Jaya, Malaysia

[4]Mechanobiology Institute, Singapore

* Correspondence: cgkoh@ntu.edu.sg; chiamkh@bii.a-star.edu.sg

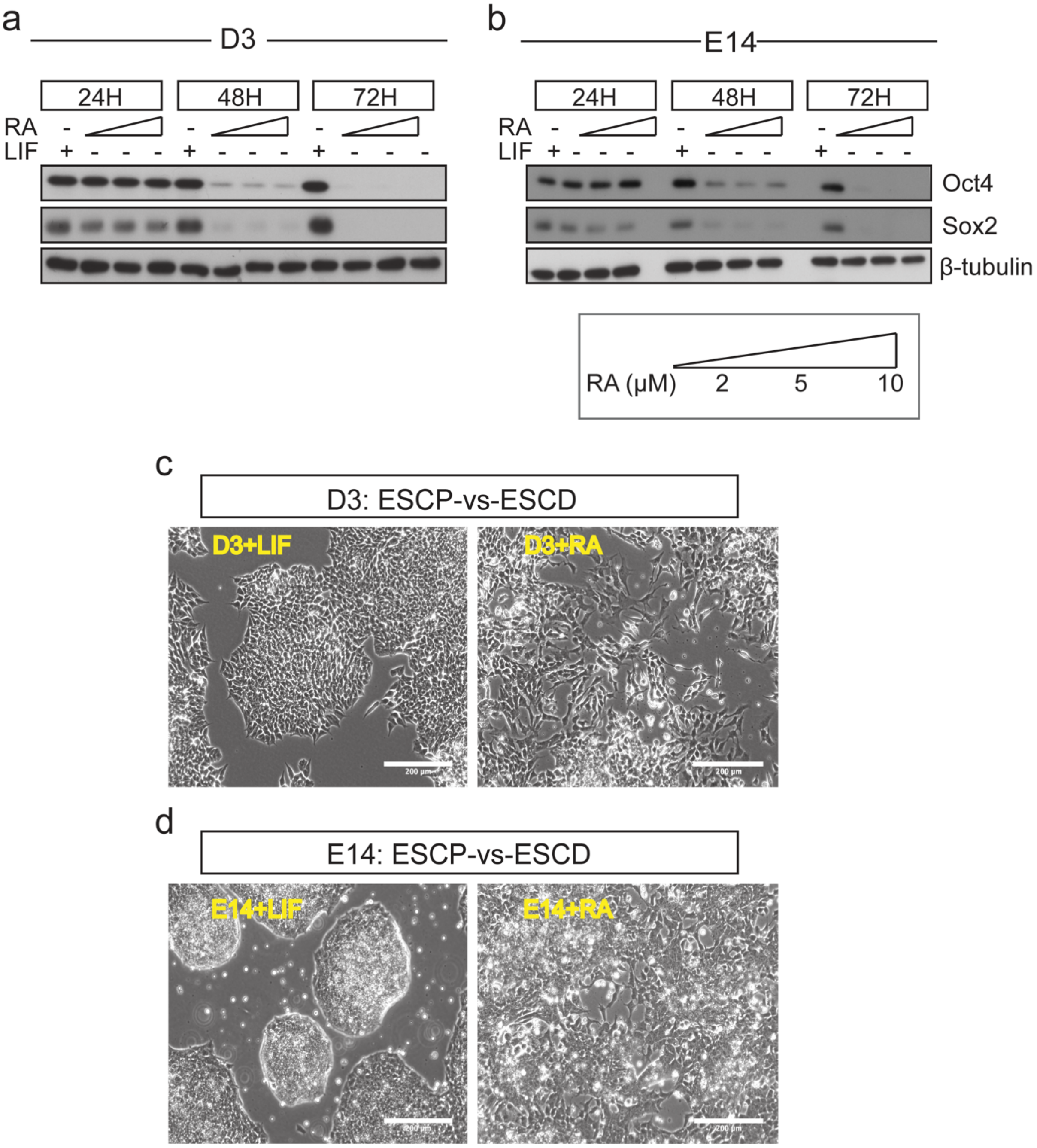

Supplementary Figure 1: Retinoic acid induces mESC differentiation. a D3 and **b** E14 cells were treated with retinoic acid for 24 hour, 48 hours and 72 hours, with increasing doses from 2 μM, 5 μM to 10 μM. Leukaemia inhibitory factor (LIF) was used to maintain pluripotency. Pluripotency markers (Oct4, Sox2) were examined by western blot. β-tubulin was used as loading control. Representative brightfield images were shown for **c** D3: ESCP-vs-ESCD and **d** E14: ESCP-vs-ESCD. Scale bar: 200 μm.

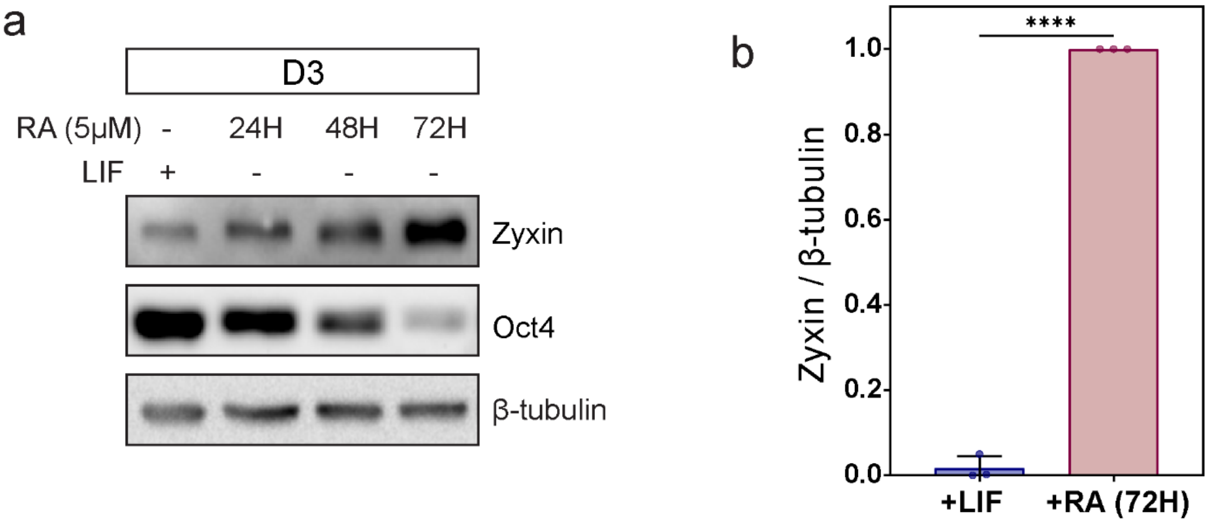

Supplementary Figure 2: Zyxin is expressed at very low level in mESCs. a To improve detection of zyxin in mESC, western blot in **Fig. 3a** was repeated without MEF protein lysates in the same gel to avoid detection errors. Increased protein loading (60 μg) and high sensitivity chemiluminescent substrates were used for detection. Optimized western blot analysis confirmed the presence of endogenous zyxin in mESCs at very low levels. Higher zyxin expression could be induced by RA treatment. **b** Zyxin intensities from D3 cells were quantified and plotted (N=3). Values were normalized against β-tubulin. Results were averaged from three biological repeats. Two-tailed unpaired Student's *t*-test was used to test the differences between +LIF and +RA. Error bars represent standard deviations. **** *$P \leq 0.0001$.*

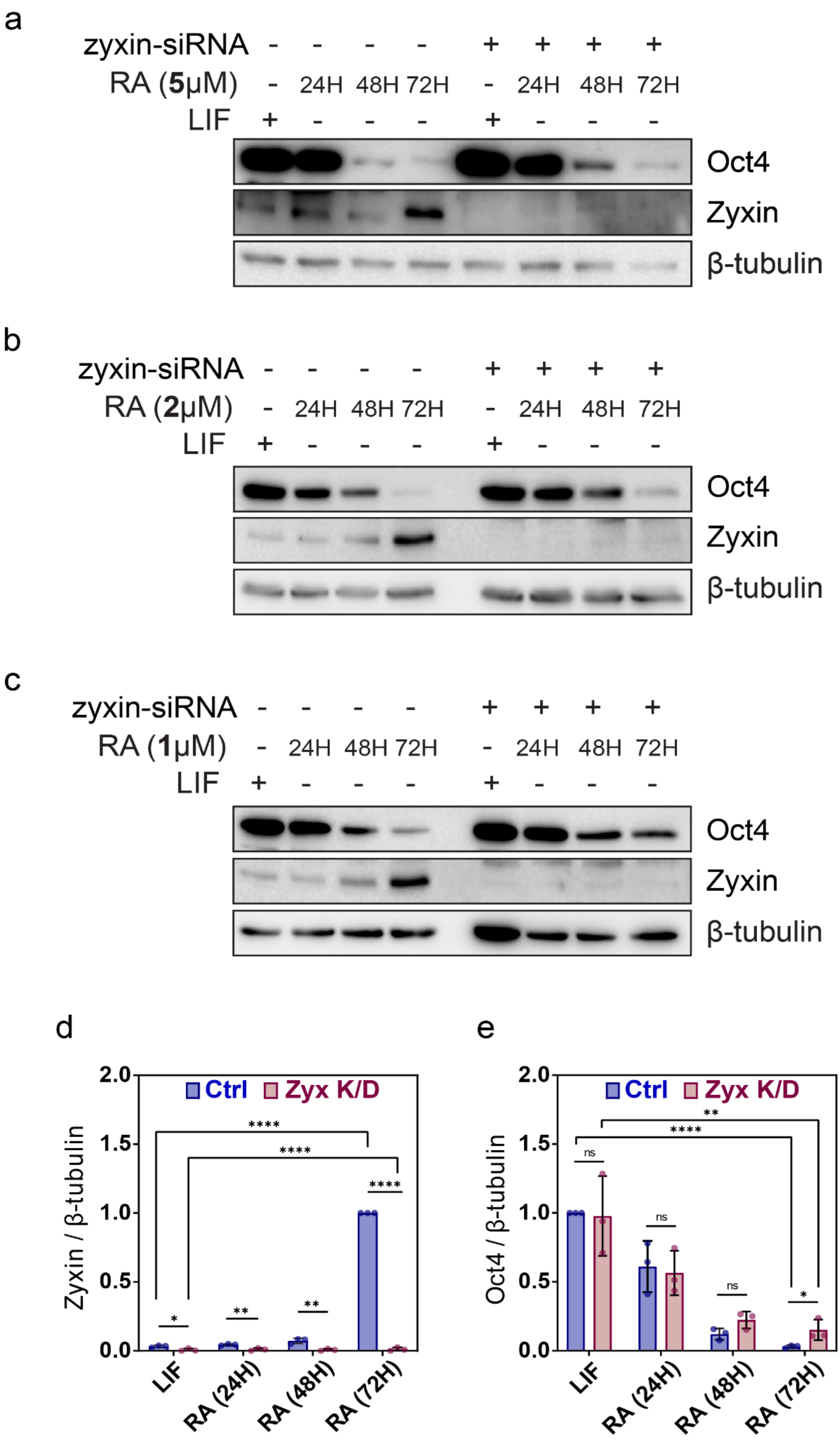
a
zyxin-siRNA - - - - + + + +
RA (5μM) - 24H 48H 72H - 24H 48H 72H
LIF + - - - + - - -
Oct4
Zyxin
β-tubulin
b
zyxin-siRNA - - - - + + + +
RA (2μM) - 24H 48H 72H - 24H 48H 72H
LIF + - - - + - - -
Oct4
Zyxin
β-tubulin
c
zyxin-siRNA - - - - + + + +
RA (1μM) - 24H 48H 72H - 24H 48H 72H
LIF + - - - + - - -
Oct4
Zyxin
β-tubulin
d
Ctrl
Zyx K/D
Zyxin / β-tubulin
2.0
1.5
1.0
0.5
0.0
****
****
****
*
**
**
LIF
RA (24H)
RA (48H)
RA (72H)
e
Ctrl
Zyx K/D
Oct4 / β-tubulin
2.0
1.5
1.0
0.5
0.0
**
****
ns
ns
ns
*
LIF
RA (24H)
RA (48H)
RA (72H)

Supplementary Figure 3: Pluripotency marker remains detectable in zyxin-knockdown cells under RA-induced differentiation. a-c Control and zyxin knockdown D3 cells were treated with decreasing concentrations of retinoic acid (RA) at (**a**) 5 μM, (**b**) 2 μM, (**c**) 1 μM. LIF is used to maintain pluripotency. Cells were lysed at 24h, 48h, 72h. Pluripotency marker (Oct4) and zyxin were examined by western blot. β-tubulin was used as loading control. **d-e** Densitometry analysis of (**d**) zyxin (normalized against β-tubulin, N=3) and (**e**) Oct4 (normalized against β-tubulin, N=3). Two-tailed unpaired Student's *t*-test was used to test the differences between LIF and RA (72H) treatment in each condition and between the control and zyxin knockdown groups within the same treatment. Results were from three independent experiments. Error bars represent standard deviations. ** $P \le 0.05$; ** $P \le 0.01$; **** $P \le 0.0001$; ns, not significant*.

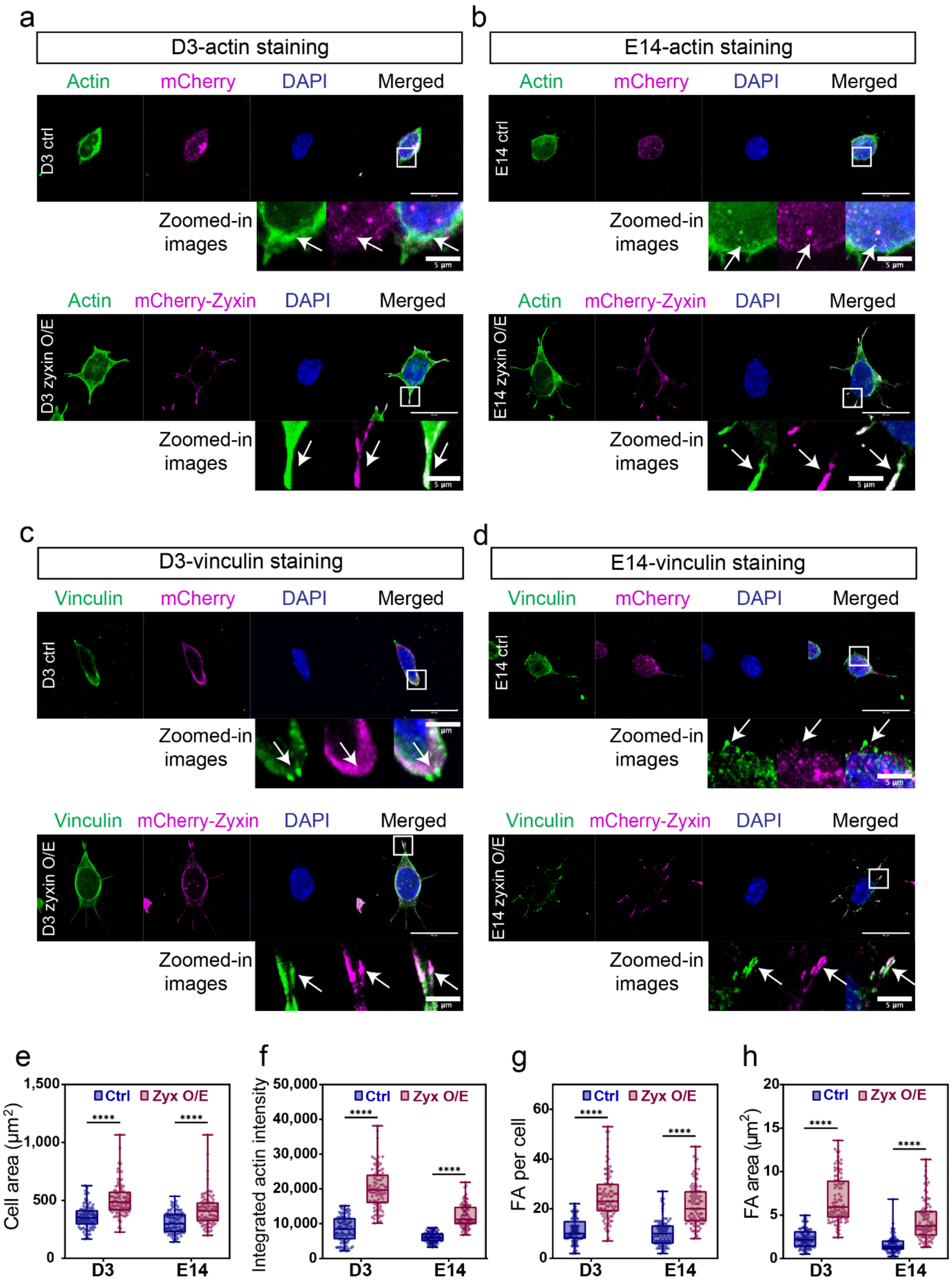

Supplementary Figure 4: Zyxin overexpression increases f-actin staining and focal adhesion numbers in cells seeded on laminin. a-d To compare stress fibres and focal adhesions, D3/E14 transfected with mCherry vector control (ctrl) and mCherry-Zyxin (zyxin O/E) were plated on laminin and immuno-stained with Phalloidin-Alexa 488 (**a-b**) and vinculin (**c-d**). White box areas were further amplified

and shown as the zoomed in images. White arrows denoted f-actin and focal adhesions. Z-stacked images with maximum intensity projection were shown. Representative images from three biological repeats were shown. Scale bar: 30 μm for original images, 5 μm for zoomed in images. **e** projected cell area, **f** integrated actin intensity, **g** number of focal adhesions per cell, **h** projected focal adhesion areas were statistically analyzed and plotted. Quantified data were from three independent experiments (N=90). Mann-Whitney *U*-test was used to test the differences between the control and zyxin overexpression groups. In the box and whisker plots, the centre line is the median, the box-bounds are the 25th and 75th percentiles, and the whiskers are the 0.05 and 0.95 percentiles. **** $P \leq 0.0001$.

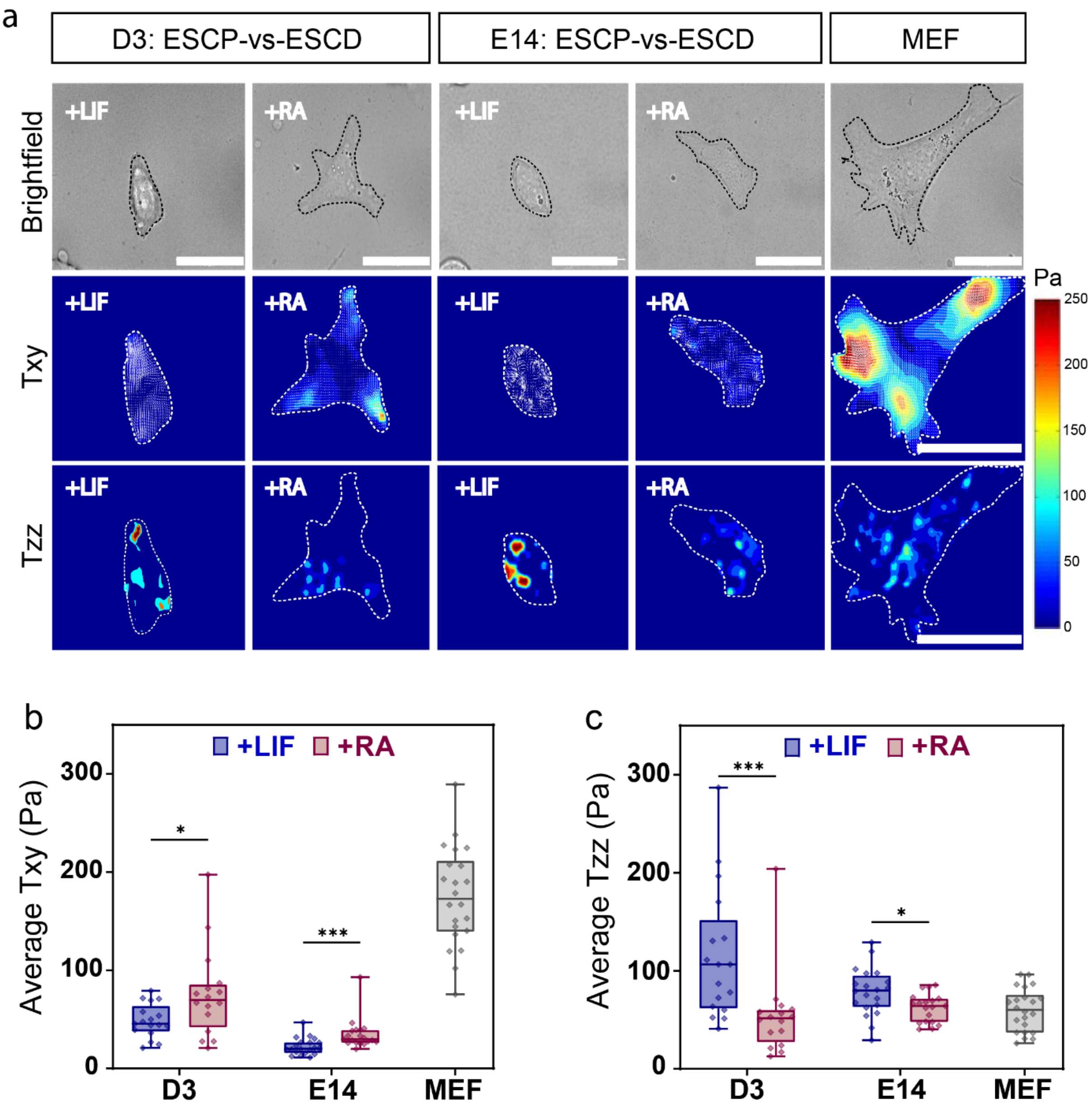

Supplementary Figure 5. Comparison of basal traction stress between pluripotent ESC (ESCP) and RA-induced differentiated ESC (ESCD). a Representative Brightfield, in-plane (Txy) traction stress images, out-plane (Tzz) traction stress images for D3, E14 and MEF were compared. **b-c** Average (**b**) in-plane (Txy) and (**c**) out-plane (Tzz) traction stress magnitudes were plotted (N=17,16,20,19,22). Mann-Whitney *U*-test was used to test the differences between the +LIF and +RA groups. In the box and whisker plots, the centre line is the median, the box-bounds are the 25th and 75th percentiles, and the whiskers are the 0.05 and 0.95 percentiles. $* P \leq 0.05$; $*** P \leq 0.001$. Scale bar: 30 μm.

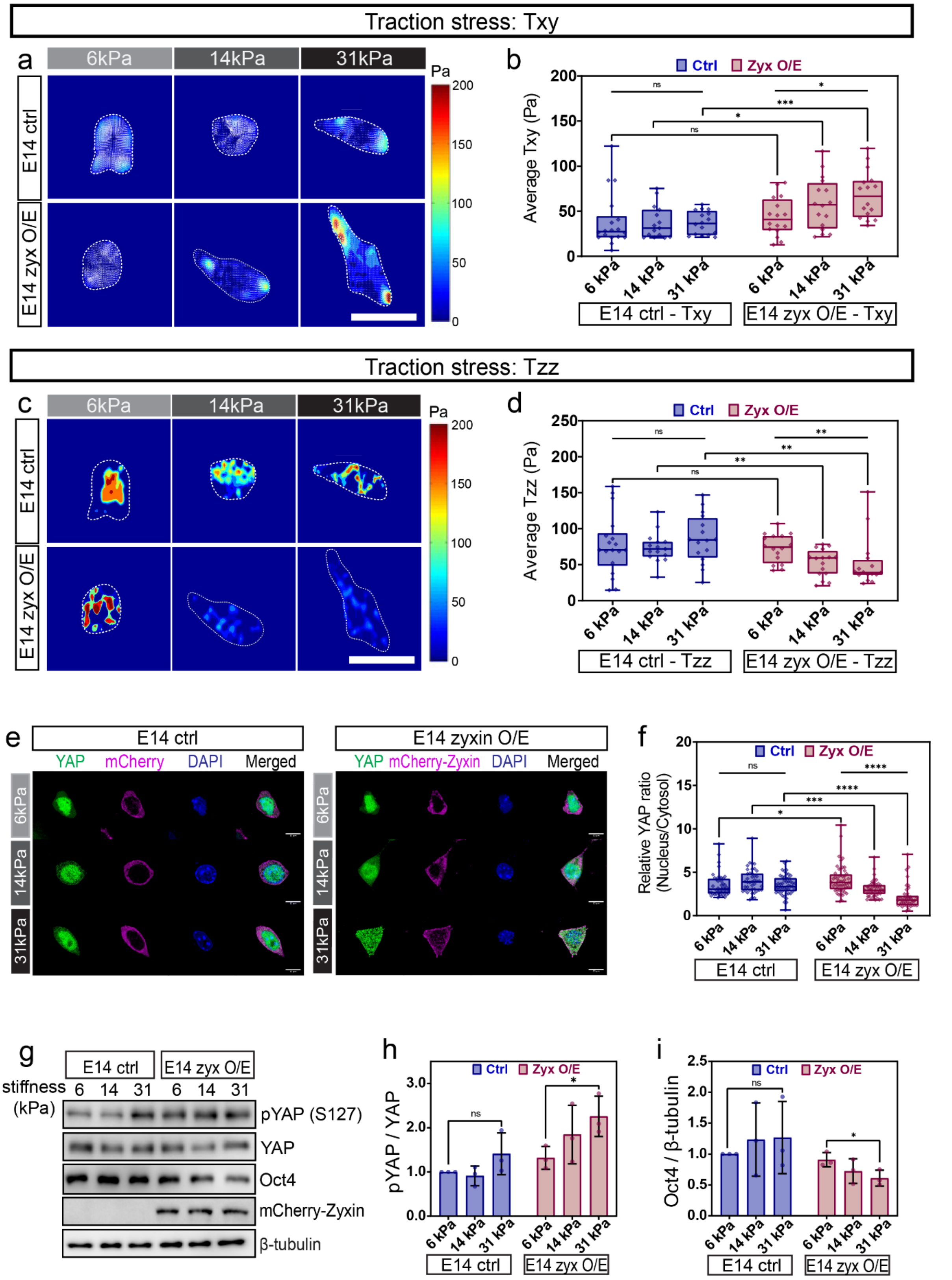

Supplementary Figure 6. Zyxin overexpression facilitates substrate rigidity sensing and inhibits YAP in E14 cell line. a Traction stress maps for x-y direction (Txy) in increasing substrate rigidity of 6 kPa, 14 kPa, 31kPa were compared between control and zyxin overexpression. **b** Quantification analysis of average traction stress

magnitudes in x-y direction (Txy) were compared between E14-control cells (E14 ctrl - Txy) (N=18,15,16) and E14-zyxin overexpressing cells (E14 zyx - Txy) (N=16,16,16). **c** Traction stress maps for z direction in increasing substrate rigidity of 6 kPa, 14 kPa, 31 kPa were compared between control and zyxin overexpression. Scale bar: 30 µm. **d** Quantification analysis of average traction stress magnitudes in z direction (Tzz) were compared between E14-control cells (E14 ctrl - Tzz) (N=18,15,16) and E14-zyxin overexpressing cells (E14 zyx - Tzz) (N=16,16,16). Kruskal-Wallis test (Ctrl - Txy, Zyx O/E – Tzz) and one-way ANOVA (Zyx O/E - Txy, Ctrl - Tzz) was used to test the differences among the three rigidities. Mann-Whitney *U*-test (Txy - 6 kPa, 14 kPa; Tzz - 31 kPa) and two-tailed unpaired Student's *t*-test (Txy - 31 kPa; Tzz - 6 kPa, 14 kPa) was used to test the differences between the control and zyxin overexpression groups at each rigidity. **e** E14 control and zyxin overexpressing cells were seeded on increasing substrate rigidity of 6 kPa, 14 kPa, 31 kPa. YAP localization was examined by immunostaining. DAPI was used to visualize the nucleus. Z-stacked images with maximum intensity projection were shown. Representative images out of three biological repeats were presented. Scale bar: 10 µm. **f** Nucleus/Cytosol YAP ratio in E14 cells transfected with either control or mCherry-Zyxin plasmids were analysed and plotted (N=44,42,49,53,47,42). Kruskal-Wallis test was used to test the differences among the three rigidities and Mann-Whitney *U*-test was used to test the differences between the control and zyxin overexpression groups at each rigidity. In the box and whisker plots, the centre line is the median, the box-bounds are the 25th and 75th percentiles, and the whiskers are the 0.05 and 0.95 percentiles. **g** E14 cells were transfected with either control or mCherry-Zyxin plasmids and then collected for western blot analysis using antibodies against pYAP, YAP, Oct4 and mCherry. β-tubulin was used as loading control. **h-i** Densitometry analysis of (**h**) pYAP (normalized against total YAP, N=3) and (**i**) Oct4 (normalized against β-tubulin, N=3). Two-tailed unpaired Student's *t*-test was used to test the differences between 6 kPa and 31 kPa in each condition. Values presented as fold change using control cells plated on 6 kPa as the internal reference. Results were from three independent experiments. Error bars represent standard deviations. ** $P \leq 0.05$; ** $P \leq 0.01$; *** $P \leq 0.001$; **** $P \leq 0.0001$; ns, not significant*.

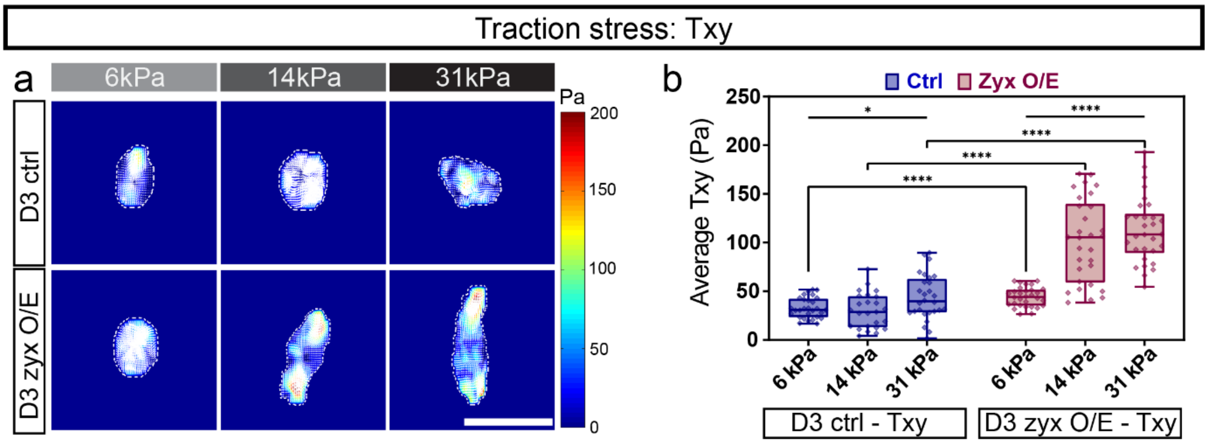

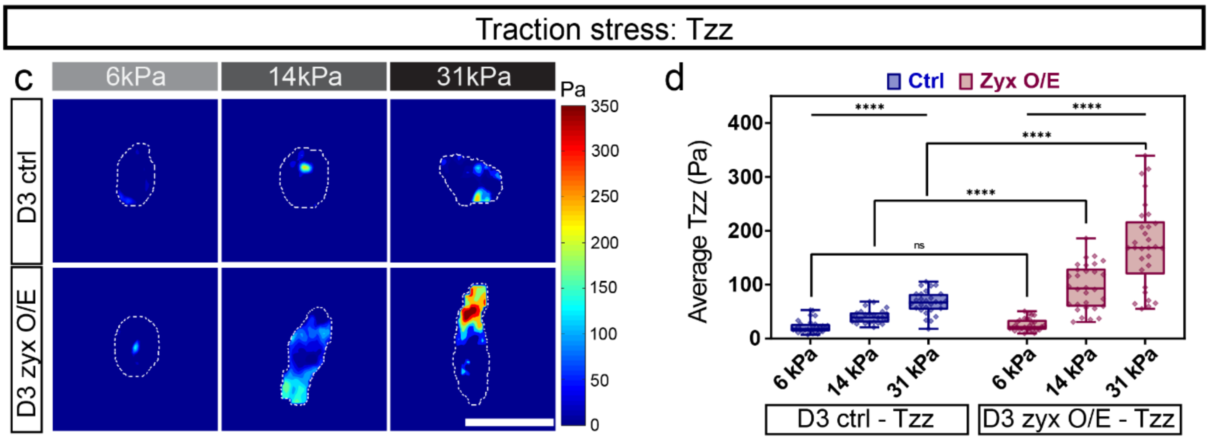

Supplementary Figure 7: Zyxin overexpression facilitates substrates rigidity sensing when cells are seeded on laminin-coated substrates. a Traction stress maps for x-y direction (Txy) in increasing substrate rigidity of 6 kPa, 14 kPa, 31 kPa were compared between control and zyxin overexpression. **b** Quantification analysis of average traction stress magnitudes in x-y direction (Txy) were compared between D3-control cells (D3 ctrl) (N=29,25,28) and D3-zyxin overexpressing cells (D3 zyx O/E) (N=30,30,30). One-way ANOVA was used to test the differences among the three rigidities. Two-tailed unpaired Student's *t*-test and was used to test the differences between the control and zyxin overexpression groups at each rigidity. **c** Traction stress maps for z-direction (Tzz) in increasing substrate rigidity of 6 kPa, 14 kPa, 31 kPa were compared between control and zyxin overexpression. **d** Quantification analysis of average traction stress magnitudes in z-direction (Tzz) were compared between D3-control cells (D3 ctrl) (N=29,25,28) and D3-zyxin overexpressing cells (D3 zyx O/E) (N=30,30,30). Kruskal-Wallis test was used to test the differences among three rigidities. Two-tailed unpaired Student's *t*-test (14 kPa, 31 kPa) and Mann-Whitney *U*-test (6 kPa) and was used to test the differences between the control and zyxin overexpression groups at each rigidity. In the box and whisker plots, the centre line is the median, the box-bounds are the 25th and 75th percentiles, and the whiskers are the 0.05 and 0.95 percentiles. ** P ≤ 0.05; **** P ≤ 0.0001; ns, not significant.* Scale bar: 30 μm.

Supplementary Table 1. Antibody and primer list.

Antibody	Species	Source	Clonal	Dilution		Catalogue number
				IF	WB	
Zyxin	Rabbit	Sigma-Aldrich, USA	Polyclonal	1:100	1:1000	Z4751
Talin	Mouse	Sigma-Aldrich, USA	Monoclonal		1:1000	T3287
Vinculin	Mouse	Sigma-Aldrich, USA	Monoclonal	1:200	1:1000	V9131
FAK	Rabbit	Upstate, USA	Polyclonal		1:500	06-543
Paxillin	Mouse	Millipore, USA	Monoclonal		1:1000	AHO0492
Oct4	Mouse	Millipore, USA	Monoclonal		1:1000	MAB4419
Sox2	Rabbit	Millipore, USA	Polyclonal		1:500	AB5603
Nanog	Mouse	Sigma-Aldrich, USA	Monoclonal		1:500	N3038
Nestin	Mouse	DSHB, USA	Monoclonal		1:250	Rat-401
Pax6	Mouse	DSHB, USA	Monoclonal		1:250	PAX6
mCherry	Rabbit	Invitrogen, USA	Polyclonal		1:500	PA5-34974
Flag	Rabbit	Sigma-Aldrich, USA	Polyclonal		1:1000	F7425
YAP	Rabbit	Cell Signaling Technology, USA	Polyclonal	1:100	1:1000	#14074
pYAP	Rabbit	Cell Signaling Technology, USA	Polyclonal		1:1000	#13008
β-tubulin	Mouse	Sigma-Aldrich, USA	Monoclonal		1:1000	T0198

Primer	Sequence (5’- 3’)
Zyxin RT-PCR primer set 1	Forward primer: CCGATGATCGAGGAACCATTC<br>Reverse primer: CGTTCTTGGTCATGTCGTCCA
Zyxin RT-PCR primer set 2	Forward primer: CAGGGAGAAAGTGTGCAGTATT<br>Reverse primer: TCGTTCTTGGTCATGTCGTCC
Oct4 RT-PCR primer	Forward primer: AGAGGATCACCTTGGGGTACA<br>Reverse primer: CGAAGCGACAGATGGTGGTC
Nanog RT-PCR primer	Forward primer: TCTTCCTGGTCCCCACAGTTT<br>Reverse primer: GCAAGAATAGTTCTCGGGATGAA

IF, Immunofluorescence
WB, Western Blot

Supplementary Figure 8: Uncropped western blots for Fig. 1a, Fig. 2a, Fig. 3a, Fig. 4a, Fig. 4b, Fig. 7d, Supplementary Fig. 1a-b, Supplementary Fig. 2a, Supplementary Fig. 3a-c, Supplementary Fig. 6g

Fig. 1a

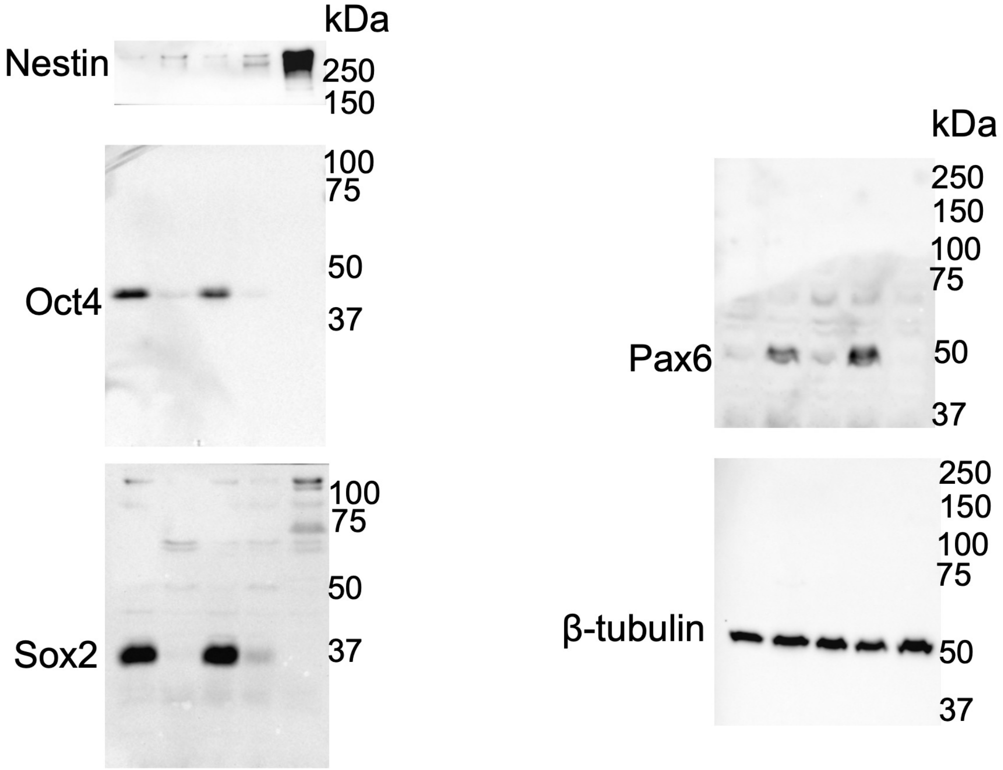

Fig. 2a

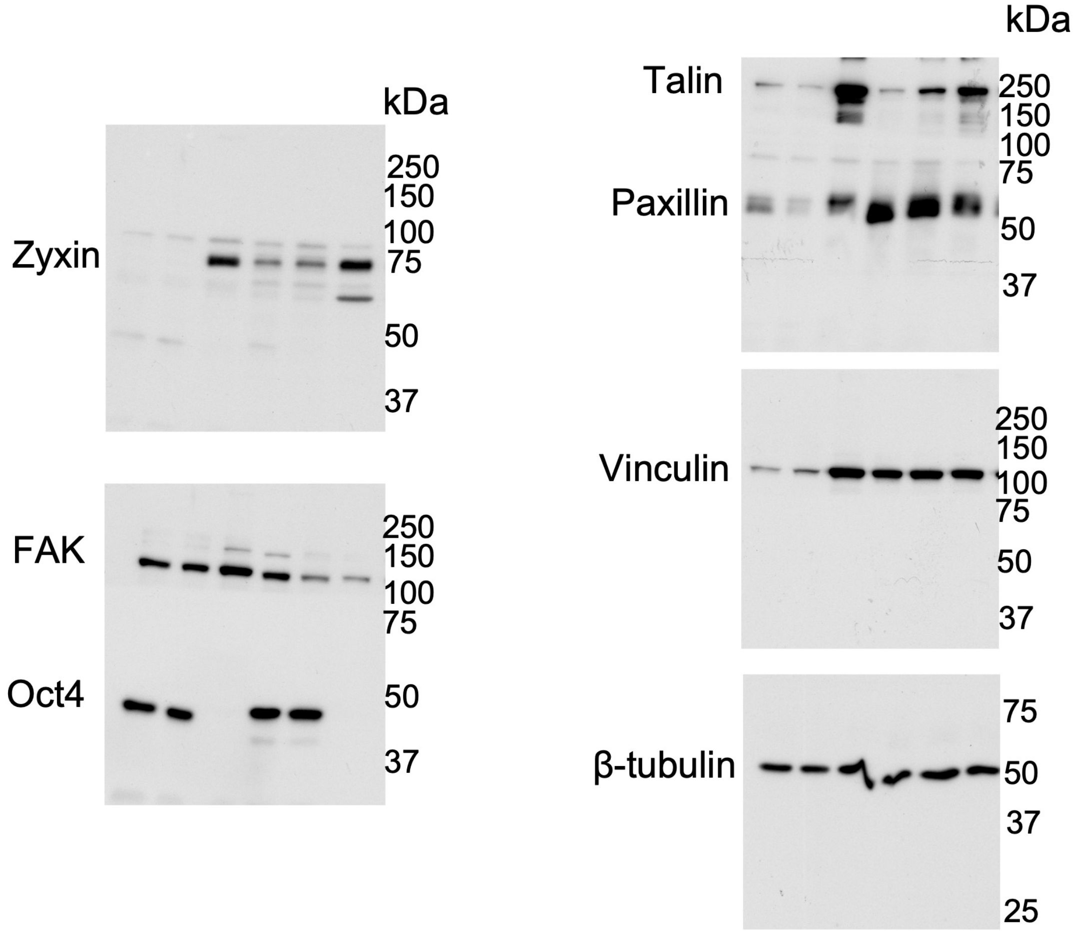

Fig. 3a

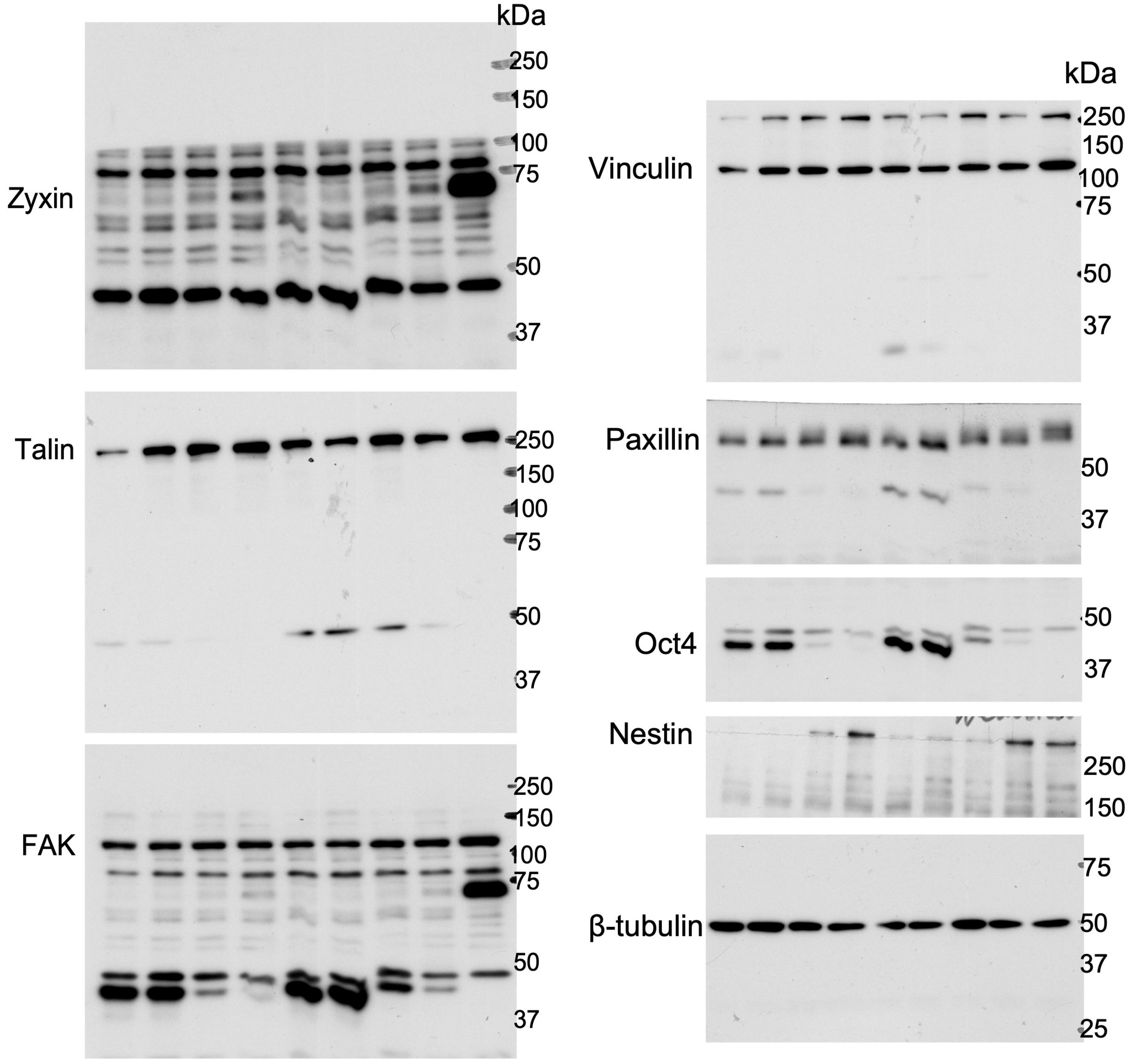
kDa
Zyxin
250
150
100
75
50
37
Talin
250
150
100
75
50
37
FAK
250
150
100
75
50
37
kDa
Vinculin
250
150
100
75
50
37
Paxillin
50
37
Oct4
50
37
Nestin
250
150
β-tubulin
75
50
37
25

Fig. 4a

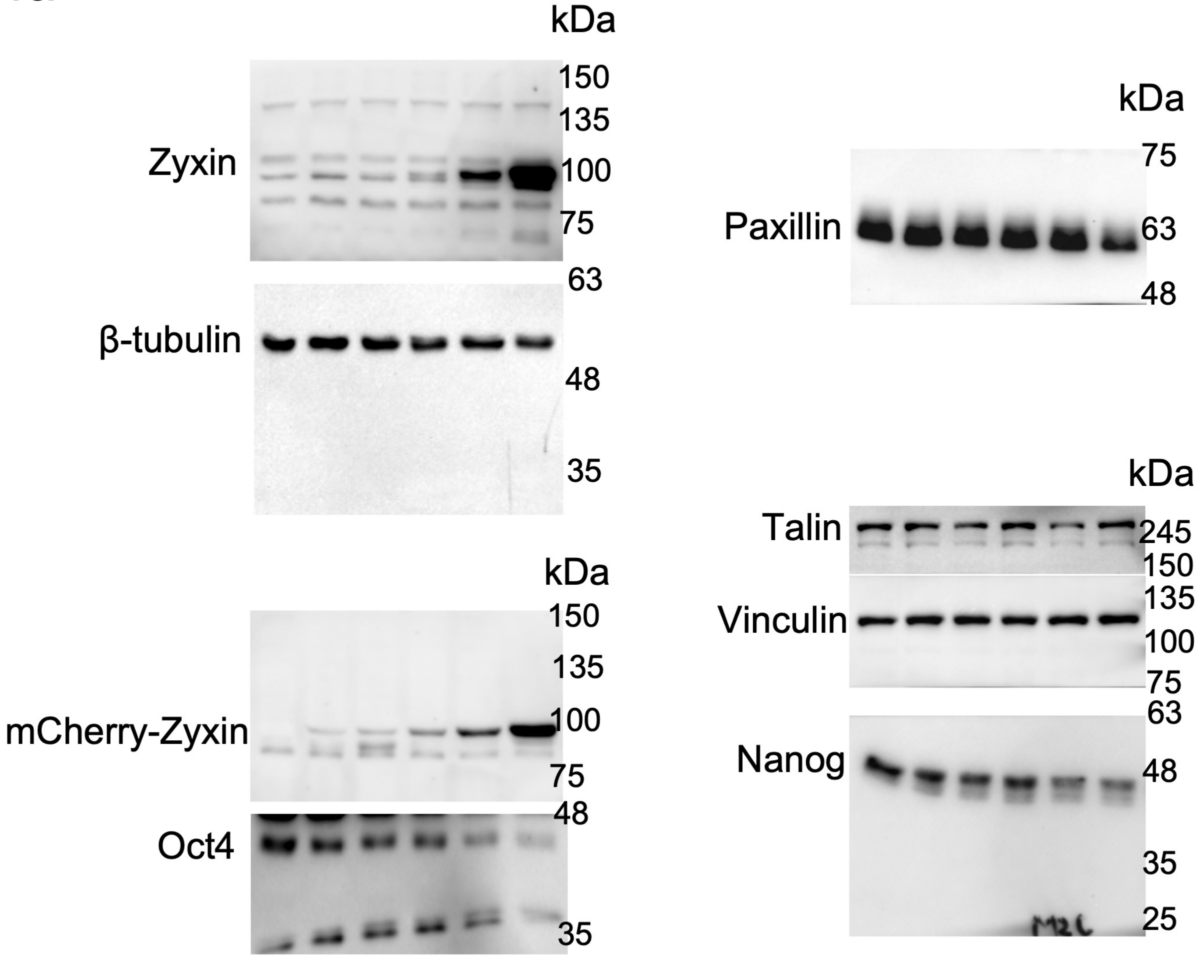
Zyxin
kDa
150
135
100
75
63
β-tubulin
48
35
mCherry-Zyxin
kDa
150
135
100
75
Oct4
48
35
Paxillin
kDa
75
63
48
Talin
kDa
245
150
Vinculin
135
100
75
Nanog
63
48
35
25

Fig. 4b

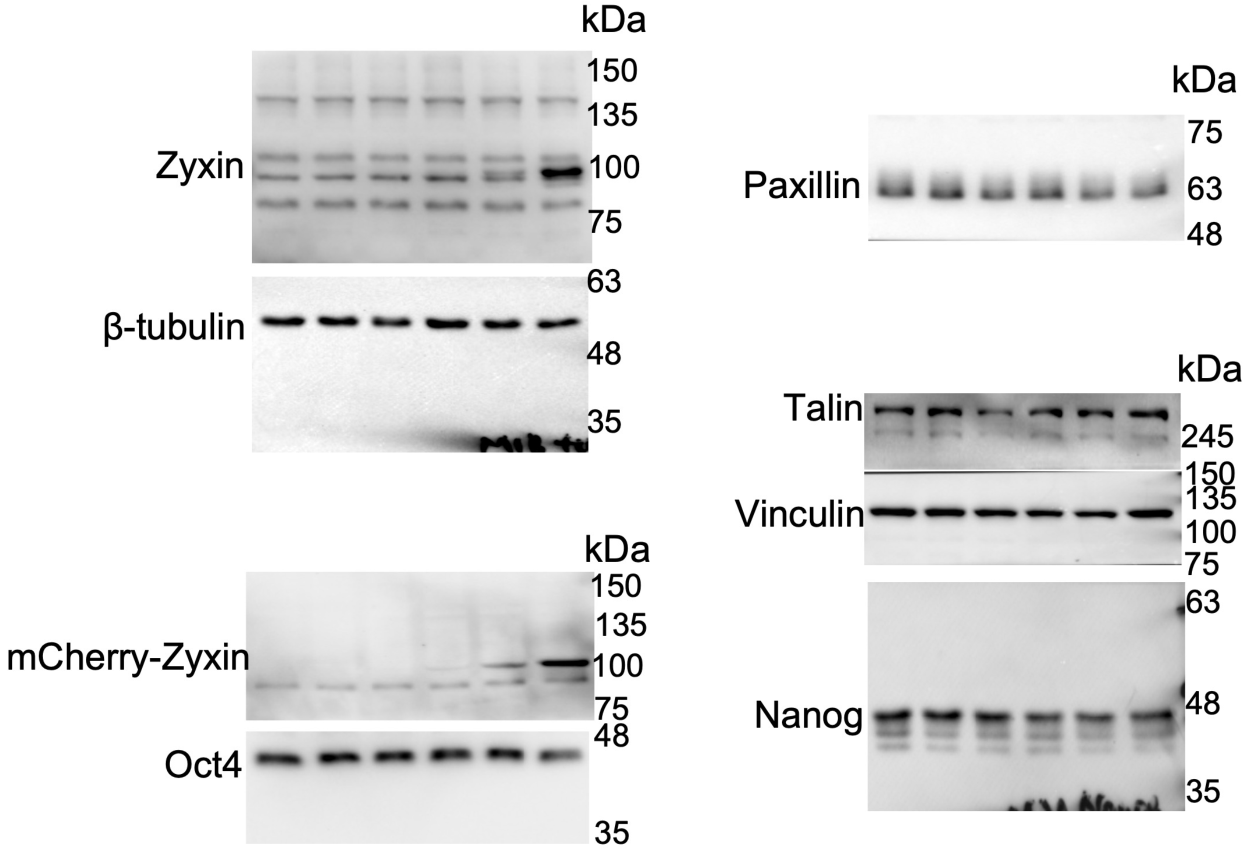
Zyxin
kDa
150
135
100
75
63
β-tubulin
48
35
mCherry-Zyxin
kDa
150
135
100
75
Oct4
48
35
Paxillin
kDa
75
63
48
Talin
kDa
245
150
Vinculin
135
100
75
Nanog
63
48
35

Fig. 7d

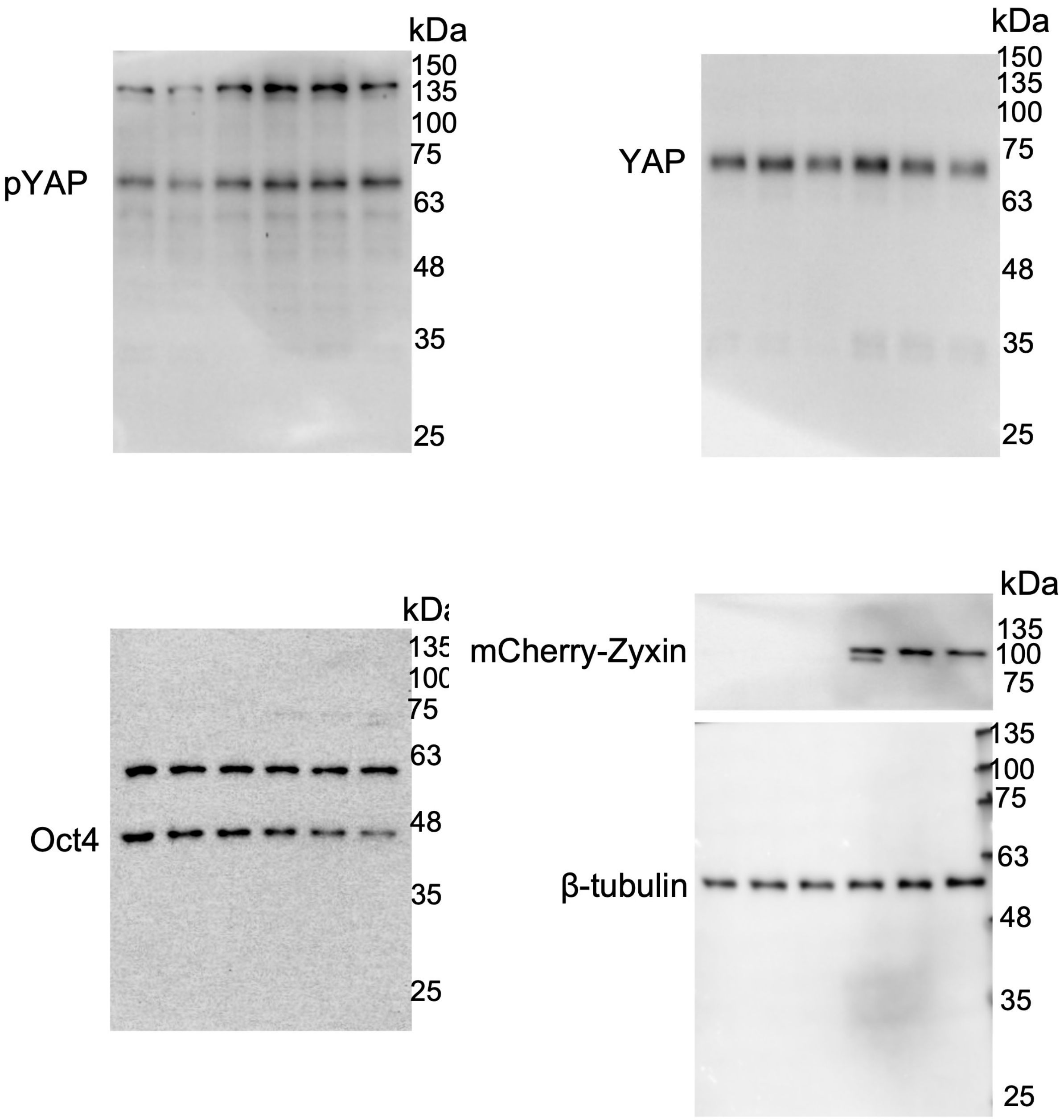

pYAP
kDa
150
135
100
75
63
48
35
25
YAP
kDa
150
135
100
75
63
48
35
25
Oct4
63
48
35
25
mCherry-Zyxin
kDa
135
100
75
β-tubulin
135
100
75
63
48
35
25

Supplementary Fig.1a-b

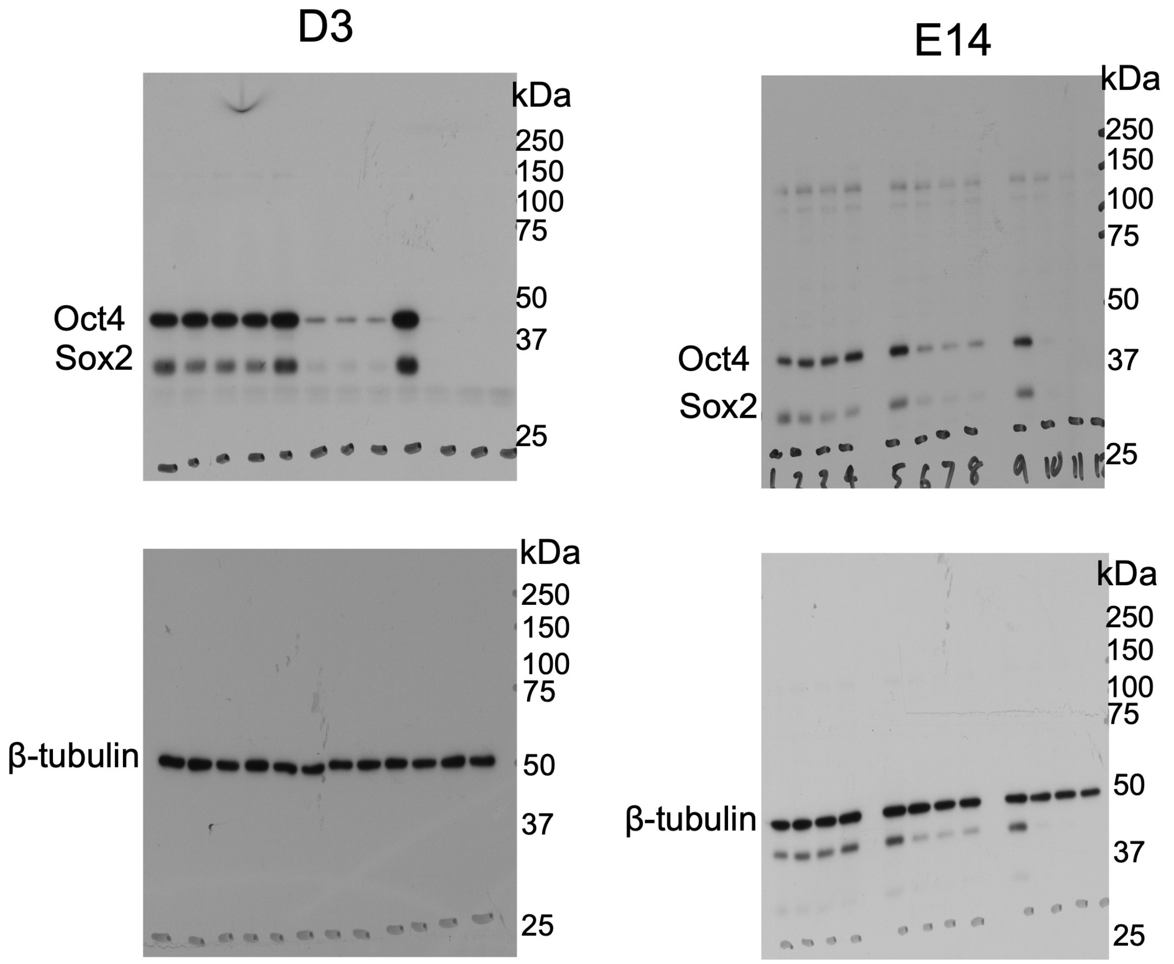

Supplementary Fig.2a

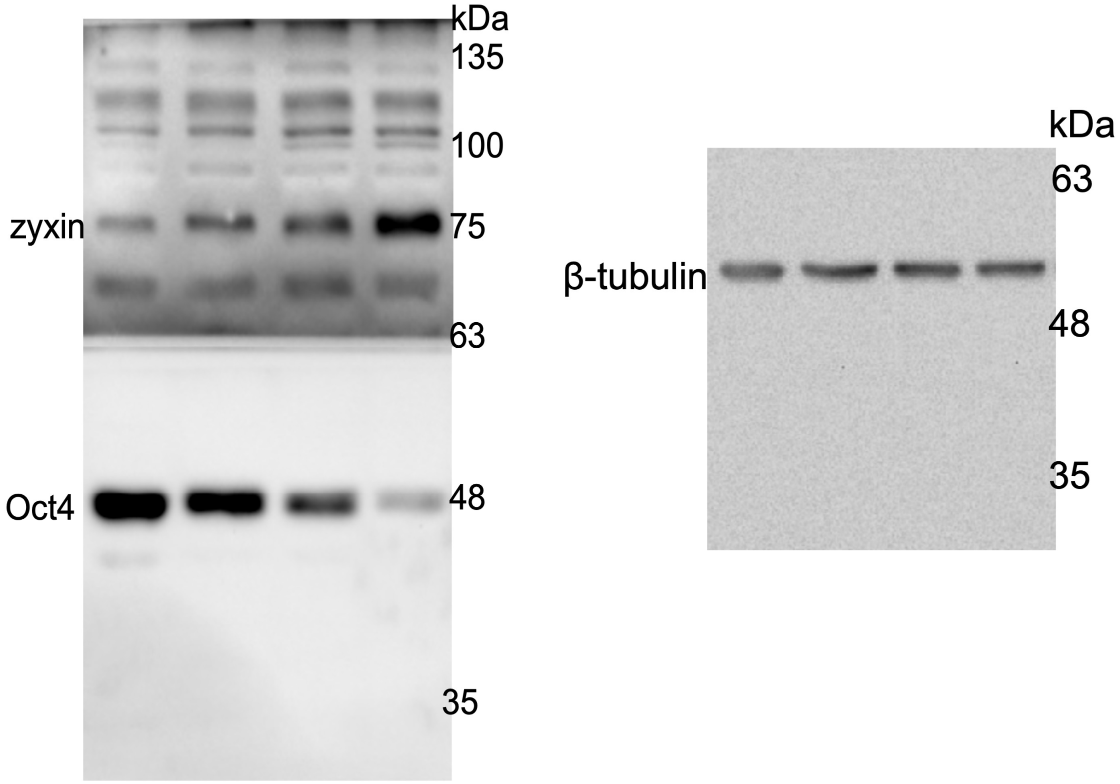

Supplementary Fig.3a-c

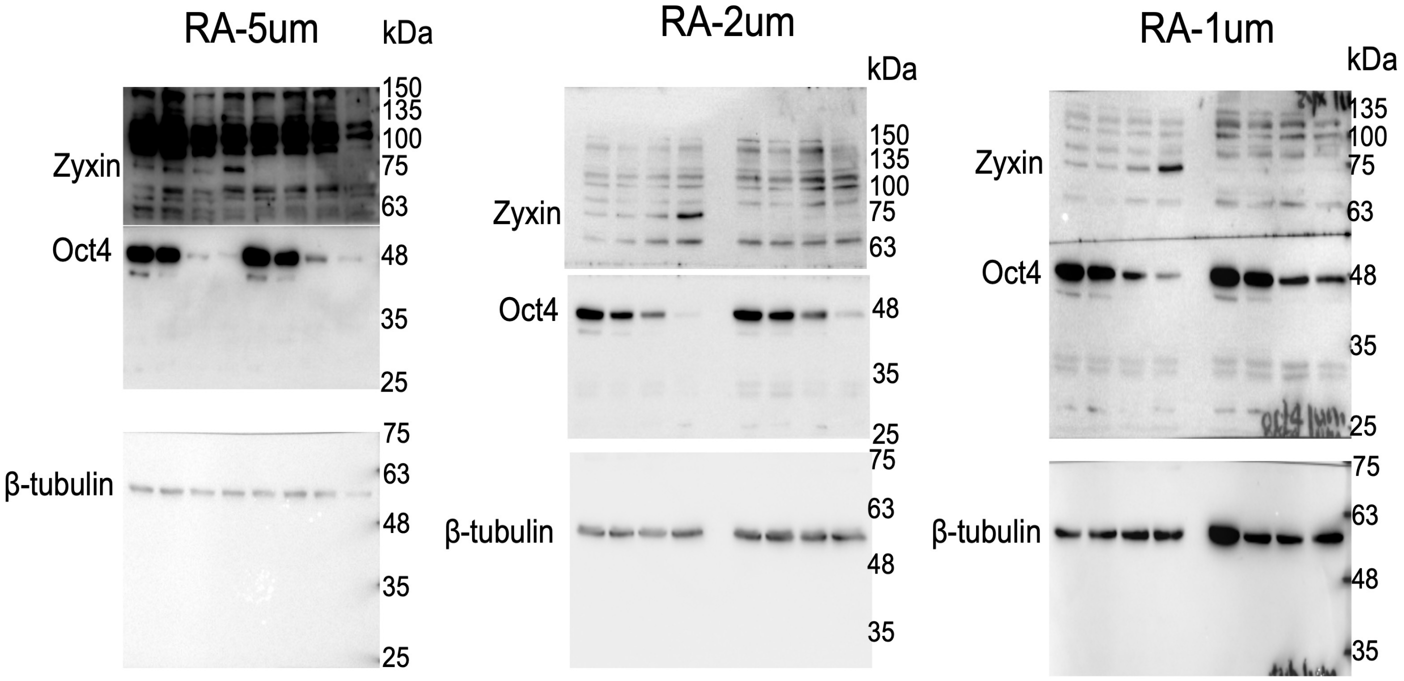

Supplementary Fig.6g

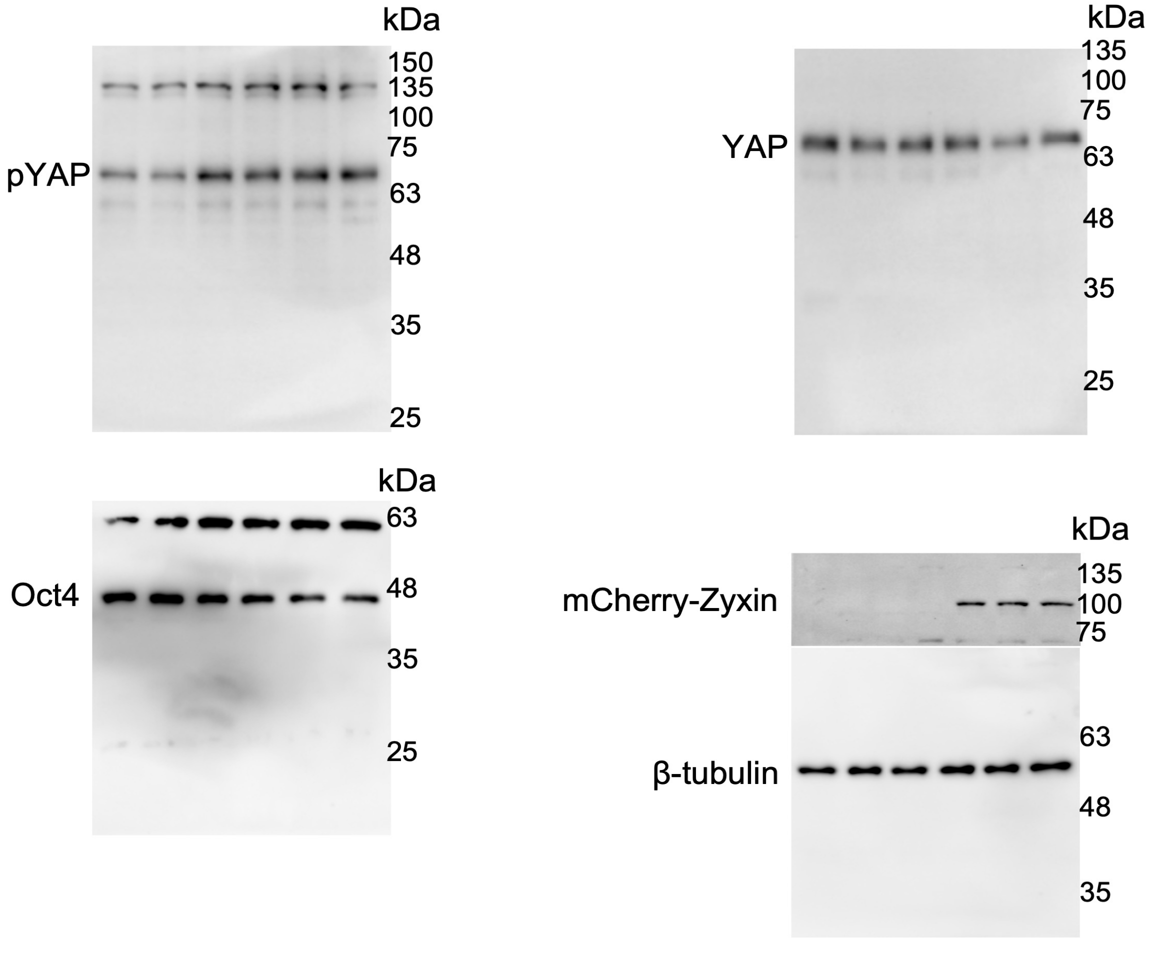

Supplement: Supplementary file 2 — Supplementary Information [file 42003_2023_4421_MOESM2_ESM.pdf]
