# Supplementary material for: Zyxin regulates embryonic stem cell fate by modulating mechanical and biochemical signaling interface
Source: Commun Biol. 2023 Jan 18;6:62. doi: 10.1038/s42003-023-04421-0 (PMC9849324; doi:10.1038/s42003-023-04421-0)
Supplement: Supplementary file 3 — Description of Additional Supplementary Files [file 42003_2023_4421_MOESM3_ESM.pdf]

Description of Additional Supplementary Files

File Name: Supplementary Data 1
Description: Source data used to generate graph for Fig. 1d,e,f,g

File Name: Supplementary Data 2
Description: Source data used to generate graph for Fig. 2b

File Name: Supplementary Data 3
Description: Source data used to generate graph for Fig. 3b,e

File Name: Supplementary Data 4
Description: Source data used to generate graph for Fig. 4c,d

File Name: Supplementary Data 5
Description: Source data used to generate graph for Fig. 5e,f,g,h

File Name: Supplementary Data 6
Description: Source data used to generate graph for Fig. 6c,d,f,h

File Name: Supplementary Data 7
Description: Source data used to generate graph for Fig. 7c,e,f

File Name: Supplementary Data 8
Description: Source data used to generate graph for Supplementary Fig. 2b

File Name: Supplementary Data 9
Description: Source data used to generate graph for Supplementary Fig. 3d,e

File Name: Supplementary Data 10
Description: Source data used to generate graph for Supplementary Fig. 4e,f,g,h

File Name: Supplementary Data 11
Description: Source data used to generate graph for Supplementary Fig. 5b,c

File Name: Supplementary Data 12
Description: Source data used to generate graph for Supplementary Fig. 6b,d,f,h,i

File Name: Supplementary Data 13
Description: Source data used to generate graph for Supplementary Fig. 7b,d
