# Supplementary material for: Zyxin regulates embryonic stem cell fate by modulating mechanical and biochemical signaling interface
Source: Commun Biol. 2023 Jan 18;6:62. doi: 10.1038/s42003-023-04421-0 (PMC9849324; doi:10.1038/s42003-023-04421-0)
Supplement: Supplementary file 17 — Reporting Summary [file 42003_2023_4421_MOESM17_ESM.pdf]

nature portfolio

Corresponding author(s): Koh Cheng Gee

Last updated by author(s): Dec 17, 2022

Reporting Summary

Nature Portfolio wishes to improve the reproducibility of the work that we publish. This form provides structure for consistency and transparency in reporting. For further information on Nature Portfolio policies, see our Editorial Policies and the Editorial Policy Checklist.

Statistics

For all statistical analyses, confirm that the following items are present in the figure legend, table legend, main text, or Methods section.

n/a	Confirmed	
☐	☒	The exact sample size (*n*) for each experimental group/condition, given as a discrete number and unit of measurement
☐	☒	A statement on whether measurements were taken from distinct samples or whether the same sample was measured repeatedly
☐	☒	The statistical test(s) used AND whether they are one- or two-sided<br>*Only common tests should be described solely by name; describe more complex techniques in the Methods section.*
☒	☐	A description of all covariates tested
☐	☒	A description of any assumptions or corrections, such as tests of normality and adjustment for multiple comparisons
☐	☒	A full description of the statistical parameters including central tendency (e.g. means) or other basic estimates (e.g. regression coefficient) AND variation (e.g. standard deviation) or associated estimates of uncertainty (e.g. confidence intervals)
☐	☒	For null hypothesis testing, the test statistic (e.g. *F*, *t*, *r*) with confidence intervals, effect sizes, degrees of freedom and *P* value noted<br>*Give P values as exact values whenever suitable.*
☒	☐	For Bayesian analysis, information on the choice of priors and Markov chain Monte Carlo settings
☒	☐	For hierarchical and complex designs, identification of the appropriate level for tests and full reporting of outcomes
☒	☐	Estimates of effect sizes (e.g. Cohen's *d*, Pearson's *r*), indicating how they were calculated

Our web collection on statistics for biologists contains articles on many of the points above.

Software and code

Policy information about availability of computer code

Data collection	ZEN blue (Carl Zeiss, Germany) software was used for images acquisition.
Data analysis	Image Lab 6.0.1 software (Bio-Rad, USA) was used to process western blot images and for bands densitometry. FIJI software (NIH, version 1.53) was used for images analysis. GraphPad Prism (version 9) was used for statistical analysis.

For manuscripts utilizing custom algorithms or software that are central to the research but not yet described in published literature, software must be made available to editors and reviewers. We strongly encourage code deposition in a community repository (e.g. GitHub). See the Nature Portfolio guidelines for submitting code & software for further information.

Data

Policy information about availability of data

All manuscripts must include a data availability statement. This statement should provide the following information, where applicable:

- Accession codes, unique identifiers, or web links for publicly available datasets
- A description of any restrictions on data availability
- For clinical datasets or third party data, please ensure that the statement adheres to our policy

All data generated during the current study were included in this article and its Supplementary Information. Un-cropped western blots accompanied by size markers were presented in Supplementary Figure 8. Description of Additional Supplementary Files was provided to describe Supplementary Data 1-13. Briefly, all source data underlying the graphs and charts were included in Supplementary Data 1-13.

Field-specific reporting

Please select the one below that is the best fit for your research. If you are not sure, read the appropriate sections before making your selection.

☒ Life sciences ☐ Behavioural & social sciences ☐ Ecological, evolutionary & environmental sciences

For a reference copy of the document with all sections, see nature.com/documents/nr-reporting-summary-flat.pdf

Life sciences study design

All studies must disclose on these points even when the disclosure is negative.

Sample size	Sample size was determined based on related published work.
Data exclusions	No data was excluded.
Replication	Three biological repeats were conducted to verify the reproducibility of the experimental findings.
Randomization	This is not relevant to our study as we do not use clinical samples.
Blinding	Investigators were blinded to data collection and data analysis.

Reporting for specific materials, systems and methods

We require information from authors about some types of materials, experimental systems and methods used in many studies. Here, indicate whether each material, system or method listed is relevant to your study. If you are not sure if a list item applies to your research, read the appropriate section before selecting a response.

Materials & experimental systems

n/a	Involved in the study	
☐	☒	Antibodies
☐	☒	Eukaryotic cell lines
☒	☐	Palaeontology and archaeology
☒	☐	Animals and other organisms
☒	☐	Human research participants
☒	☐	Clinical data
☒	☐	Dual use research of concern

Methods

n/a	Involved in the study	
☒	☐	ChIP-seq
☒	☐	Flow cytometry
☒	☐	MRI-based neuroimaging

Antibodies

Antibodies used

Antibody, Species, Source, Clonal, Catalog number
Zyxin, Rabbit, Sigma-Aldrich USA, Polyclonal, Z4751
Talin, Mouse, Sigma-Aldrich USA, Monoclonal, T3287
Vinculin, Mouse, Sigma-Aldrich USA, Monoclonal, V9131
FAK, Rabbit, Upstate USA, Polyclonal, 06-543
Paxillin, Mouse, Millipore USA, Monoclonal, AHO0492
Oct4, Mouse, Millipore USA, Monoclonal, MAB4419
Sox2, Rabbit, Millipore USA, Polyclonal, AB5063
Nanog, Mouse, Sigma-Aldrich USA, Monoclonal, N3038
Nestin, Mouse, DSHB USA, Monoclonal, Rat-401
Pax6, Mouse, DSHB USA, Monoclonal, PAX6
mCherry, Rabbit, Invitrogen USA, Polyclonal, PA5-34974
Flag, Rabbit, Sigma-Aldrich USA, Polyclonal, F7425
YAP, Rabbit, Cell Signaling Technology USA, Polyclonal, #14074
pYAP, Rabbit, Cell Signaling Technology USA, Polyclonal, #13008

Validation

All antibodies used in the study were validated on the manufacturer's website with relevant citations or immunoblotting and immunostaining supporting data.

Eukaryotic cell lines

Policy information about cell lines

Cell line source(s)	All cell lines used were sourced from ATCC.
Authentication	None of the cell lines used were authenticated.
Mycoplasma contamination	All cell lines were tested negative for mycoplasma.
Commonly misidentified lines (See ICLAC register)	No misidentified line was used in this study.
